# CAR T‐Cell Therapies for Patients With Relapsed and Refractory Aggressive Lymphomas: Real‐World Experiences From a Single Center on the Use of Radiotherapy

**DOI:** 10.1002/hon.70124

**Published:** 2025-08-31

**Authors:** J.T. Jutzi, J. Wampfler, C. Ionescu, M.N. Kronig, B. Jeker, M. Hoffmann, I. Reusser, C. Haslebacher, S. Sendi Stamm, M. Schletti, B.P. Lüscher, V.U. Bacher, M. Wehrli, M. Daskalakis, T. Pabst, U. Novak

**Affiliations:** ^1^ Department of Medical Oncology Inselspital Bern University Hospital University of Bern Bern Switzerland; ^2^ Department of Radiation Oncology Inselspital Bern University Hospital University of Bern Bern Switzerland; ^3^ Department of Hematology and Central Hematology Laboratory Inselspital Bern University Hospital University of Bern Bern Switzerland

**Keywords:** CAR T‐cells, lymphomas, radiotherapy, real‐world

## Abstract

In this retrospective analysis on patients treated with CAR T‐cells at our center, we report on the use of radiotherapy in this setting. Our real‐world cohort of 90 patients with aggressive lymphomas was treated with CARs from 2019 until December 2022. We found that the outcome of a localized relapse after CARs treated with radiotherapy was comparable to patients without a relapse. With the knowledge from the collected real‐world data, we should launch prospective clinical trials to further improve the use of radiotherapy, and overall the efficacy of CAR T‐cell therapies for patients with aggressive lymphomas.

Chimeric antigen receptor (CAR) T‐cell therapy (CARs) is a standard therapy for hematological malignancies, with a curative potential in relapsed aggressive B‐cell lymphomas. Radiotherapy (RT) is a locally effective and readily available therapy that can be used before or as a bridging after lymphapheresis, or as salvage to treat relapses after CAR infusion. RT is particularly useful for symptomatic manifestations [1]. However, its exact role and impact in the setting of CAR T‐cell therapies is currently unclear and evolving by its use in the real‐world.

136 patients received CAR T‐cell therapies from 2019 until December 2022 with different products and indications at the Inselspital/Bern University Hospital. The study was approved by the local ethics committee (No. 2018–00628 and 2022–00711), and all patients signed informed consent and/or did not declare refusal to participate. We here report on the use of RT in 90 patients (66.2%) with aggressive lymphomas, mainly DLBCL and high‐grade lymphomas, including 12 patients with secondary CNS manifestations, and 24 with transformed lymphomas. They received either Kymriah (*n* = 55), Yescarta (*n* = 29), or Liso‐Cel (*n* = 6, as part of a clinical trial) after two or more previous lines of therapy, the approval status in Switzerland during this period. The median age of the cohort is 67 years (range 35–82 years). 27 patients (30%) received RT when the process toward a CAR‐T cell therapy was initiated with the reimbursement request, 6 patients received RT before lymphapheresis, 7 as part of the bridging before, and 14 after CAR infusion to treat a relapse. One patient received RT before lymphapheresis and for bridging. Data cutoff for the survival is march 2023.

To control the disease and related localized symptoms, as expected, RT was applied to various localizations: before lymphapheresis to (abdominal) bulky disease (with 36 and 40 Gy, in 2 patients) and various nodal sites (with 10–46 Gy, 4 patients). Bridging RT was given to manifestations in the bone, brain and lymph nodes (10–36 Gy, 7 patients). The median time of the end of radiotherapy to lymphapheresis was 29 days (range 1–58), and 25 days (range 10–63) before CAR infusion when RT was given as the bridging option. When given before lymphapheresis, the overall response rate (ORR) of radiotherapy in our cohort was 60%, including one complete and two partial responses. We think that given the subsequent infusion of CAR T‐cells after bridging and the overall timing, the efficacy of RT as a bridging therapy in terms of its response alone cannot be reported separately in our cohort.

A lymphoma relapse was detected in 32/90 patients (35.5%), that was either biopsy proven (14/32, 43.8%) or the latter was based on imaging (PET or MRI) or symptoms in the remaining patients. The relapses occurred at a median of 157 days (range 38–568) after CAR infusion. 14/32 (43.8%) of these patients received salvage RT; in 11 patients (79%), with isolated or localized lesion, as a newly established institutional policy, they received RT as a comprehensive therapy with curative intent. The lesions were mostly in lymph nodes (7 patients), or were localized in the breast, brain, sinuses and bone (one patient each), and were treated with doses ranging from 19.5 to 46 Gy. The ORR was 50%, with 5 CRs and 2 PRs; two patients could not be evaluated for response. We compared the survival of these patients with patients without a relapse after CARs. Interestingly, a similar outcome was found, confirming the rational of our approach (Figure 1).

**FIGURE 1:**
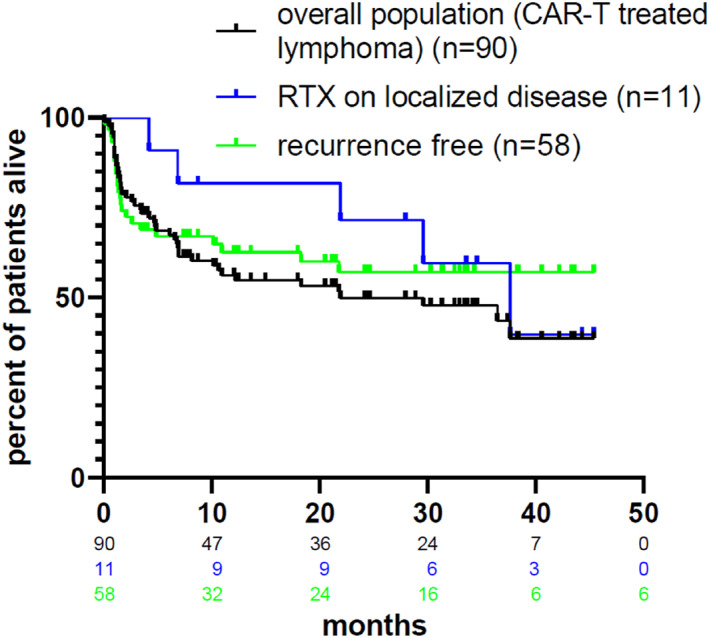
Effect of radiotherapy for patients with aggressive lymphomas treated with CAR T‐cells.

We previously reported a number of real‐world observations for lymphoma patients treated with CARs [2, 3, 4, 5]. Several limitations of this study should be acknowledged. The single‐center, retrospective design introduces inherent biases and may limit the generalizability of the findings. Second, the heterogeneity in the administration and the use of RT with an evolving role over time and perceived effect since CARs were applied needs to be taken into account. However, we are convinced that clinically relevant conclusions can be drawn. In the context of CAR‐T cell therapies, the current role of RT is to control the disease and to limit the toxicity of CARs by debulking. However, interestingly, in a mouse model, radiotherapy was shown to enhance CAR‐T efficacy [6]. This priming role during bridging to overcome treatment resistance and improve clinical outcomes is under investigation in prospective clinical trials and different techniques (NCT05574114) [7, 8]. A third of our lymphoma patients received radiotherapy, which is more than we used in the relapsed setting before the availability of CARs. In our cohort of patients that was chosen based on a pretreatment with a minimum of two previous lines of systemic therapy, radiotherapy was given before and after CAR infusion and as a bridging therapy. As part of the “brain‐to‐vein time” [9], radiotherapy as a salvage ideally complements the time‐sensitive systemic options likely without a negative impact on the lymphapheresis product [10, 11]. Given its prompt availability without a reimbursement request beforehand, an important aspect in the legal context of Switzerland, and its rapid effectiveness, it is particularly useful to treat symptomatic manifestations. Its locally limited effect can be offset by the subsequent systemically active CARs which is important in the bridging phase [10, 12, 13]. With the use of a comprehensive RT to a localized relapse after CAR T‐cell therapy, we found an outcome that is comparable to CAR T‐cell treated patients without a relapse (Figure 1), confirming reports showing an up to 70% in‐field disease control [14, 15]. In our cohort of patients, we used radiotherapy from a practical point of view, and are well aware of additional conceptual aspects and assumed synergies that may expand its future use [1].

*In summary*, we confirm the effectiveness of radiotherapy in the curative therapy complex of CAR T‐cell therapies for patients with aggressive lymphomas. Specifically, we found that the outcome of a localized relapse after CARs treated with radiotherapy was comparable to patients without a relapse, defining a possible standard for these patients.

## Conflicts of Interest

J.W. reports on advisory board participations with Gilead and Novartis, AstraZeneca, Daiichi Sankyo, ExactSciences, GSK, MSD and Sanofi, and received travel support from AstraZeneca and MSD. M.W. reports on consulting fees and advisory board participation to/through the institution from and with Gilead, Pierre Fabre, LUMICKS, Miltenyi and Celgene (BMS). M.D. reports support for travel, accommodation and expenses from Kite‐Gilead, Novartis, Amgen, Novo Nordisk, and consulting or advisory roles for Novartis, Alexion Pharma. T.P. was involved in advisory boards of BMS, Kite Gilead, Novartis and Janssen. U.N. reports consulting fees and advisory board participation to/through the institution from and with Janssen‐Cilag, Celgene (BMS), Takeda, AstraZeneca, Roche, Novartis, Incyte, Beigene, Kyowa Kirin, Gilead, Pierre Fabre and Miltenyi, payment of honoraria to the institution form Celgene (BMS), Novartis, Takeda, and Gilead, and meeting and/or travel support to the institution from Janssen, Roche, Gilead and Takeda. All other authors declare no conflicts of interest.

## Peer Review

The peer review history for this article is available at https://www.webofscience.com/api/gateway/wos/peer-review/10.1002/hon.70124.

## Data Availability

The data that support the findings of this study are available on request from the corresponding author. The data are not publicly available due to privacy or ethical restrictions.
